# Supplementary material for: Respiratory infection transmission risk and indoor air quality at outpatient departments and emergency treatment units of Sri Lankan teaching hospitals
Source: PLOS Glob Public Health. 2024 Feb 26;4(2):e0002862. doi: 10.1371/journal.pgph.0002862 (PMC10896534; doi:10.1371/journal.pgph.0002862)
Supplement: Attachment — Submitted filename: Comments.docx [file pgph.0002862.s002.docx]

The article Titled “ Respiratory Infection Transmission Risk and Indoor Air Quality at Outpatient Departments and Emergency Treatment Units of Sri Lankan Teaching Hospitals” is a good attempt to evaluate the air quality in hospitals and medical care facilities. However in its present form it looks a little jumbled up and the authors need to sort out the sectioning to bring out the clarity. Furthermore, the following comments must also be addressed to enhance the quality of the article

Abstract:

1. Define abbreviations

2. Define all the measured parameters.

3. Which particular volatile organic compounds were measured?

4. Why COVID-19 is mentioned as a keyword, although its nowhere mentioned in the abstract.

**Introduction**

1. There is no need to define CO2 again

2. The introduction is long and disjointed. The authors must make it short and relevant to the theme of the article.

**Material and Methods**

1. On which basis the hospital selection was done

2. Instrumentation is not adequate

3. The sample size was not adequate.

**Results and Discussion**

1. Some higher order statistics must be implemented to represent the results

2. Correlate the findings with the previous literature.

3. The health risk assessment of these pollutants must be done.
