# Supplementary material for: Respiratory infection transmission risk and indoor air quality at outpatient departments and emergency treatment units of Sri Lankan teaching hospitals
Source: PLOS Glob Public Health. 2024 Feb 26;4(2):e0002862. doi: 10.1371/journal.pgph.0002862 (PMC10896534; doi:10.1371/journal.pgph.0002862)
Supplement: Attachment — Submitted filename: Minor Revisions.docx [file pgph.0002862.s003.docx]

Review of the paper titled *“Respiratory Infection Transmission Risk and Indoor Air Quality at Outpatient Departments and Emergency Treatment Units of Sri Lankan Teaching Hospitals”.*

1. L-193. Use the word “measurements” instead of the word readings and replace the word readings with measurements throughout the manuscript.
2. L 256-257, Provide the reason for high levels of pollutants since it is mentioned only as unusually high. Give the reason for these unusually high levels of pollutants.
3. L 333 – 335, check the grammar and rewrite the sentence providing the same meaning.
4. L 386-390, Provide the reason for high CO2 Levels.
5. L 394 – Check grammar and rewrite the sentence.

Although, the paper is discussing the indoor CO2 levels which will serve as a proxy for representing the ventilation levels, a simple calculation of Air changes per hour (ACH) in hospitals with manual ventilation would provide a strong foundation for designing efficient ventilation systems.
