# Supplementary material for: Respiratory infection transmission risk and indoor air quality at outpatient departments and emergency treatment units of Sri Lankan teaching hospitals
Source: PLOS Glob Public Health. 2024 Feb 26;4(2):e0002862. doi: 10.1371/journal.pgph.0002862 (PMC10896534; doi:10.1371/journal.pgph.0002862)
Supplement: Attachment — Submitted filename: Response to Reviewers-R1-Nov2023.docx [file pgph.0002862.s004.docx]

**PGPH-D-23-01122
Respiratory Infection Transmission Risk and Indoor Air Quality at Outpatient Departments and Emergency Treatment Units of Sri Lankan Teaching Hospitals.
PLOS Global Public Health**

**The rebuttal letter that responds to each point raised by the editor and four reviewers- R1.**

Dear Dr. Hanna Landenmark,

Thank you for the email dated Nov6, 2023.

The extensively revised clean file version of the manuscript and the marked-up version with alterations highlighted are also submitted with this.

To enhance clarity, our responses are in green color fonts.

We hope we have satisfactorily addressed all the comments/concerns reasonably and hope that our paper may now be suitable for publication in your journal.

Dr.NDB Ehelepola (Corresponding author)

……………………………………………….

**Editors Concerns**

Please see the comments of four reviewers below and in the attachment. All reviewers seem positive about the contributions of the study, and have suggested revisions to strengthen the study.

Please note that reviewer 1's comments about figures being blurry is only a result of the generation of the reviewer PDF, and that the original resolution in which the figures are uploaded is fine, so there is no need to enhance these figures. Please ensure that no figures are copyrighted, and please insert information on how the figures were created, and by whom, in the figure legend, and whether any base maps or stock figures were used.

Implemented. Now there is a striking image and 3 figures. We mentioned this explanation in response to reviewer#1 concerns of figures being blurry. Kindly see figure legends for the information added.

Striking image: Indoor air quality (IAQ) parameters we studied. This figure was created by the first author using Microsoft Publisher 2010 software and associated clipart and clipart from https://www.freeimages.com/clipart were used.

Figure1: Schematic representation of how respiratory infections are transmitted. This figure was created by the first author using Microsoft Publisher 2010 software and associated clipart and clipart from <https://www.freeimages.com/clipart>.

Figure2: The locations of teaching hospitals of Sri Lanka participated in this study are marked as blue dots in the map. The background shows population density in color codes. Due to the proximity of some hospitals to each other, some blue dots overlap at this scale. We adopted Figure1 of https://doi.org/10.1371/journal.pntd.0004813 published under CC BY4.0. [35] as the base layer of this map.

Figure 3: Average CO2 concentrations of OPDs and ETUs of each hospital. This figure was created by the first author using Microsoft Office 2010 software.

Journal Requirements:

Please provide separate figure files in .tif or .eps format.

Implemented. Figures were PACE engine checked.

.

Potential Copyright Issues:

Figure 3: please (a) provide a direct link to the base layer of the map (i.e., the country or region border shape) and ensure this is also included in the figure legend; and (b) provide a link to the terms of use / license information for the base layer image or shapefile. We cannot publish proprietary or copyrighted maps (e.g. Google Maps, Mapquest) and the terms of use for your **map base layer must be compatible with our CC-BY 4.0 license**.

Implemented. Former figure 3 is now figure 2. The figure base was a figure created under CC-BY 4.0 license. That source was acknowledged. New figure legend: *Figure2: The locations of teaching hospitals of Sri Lanka participated in this study are marked as blue dots in the map. The background shows population density in color codes. Due to the proximity of some hospitals to each other, some blue dots overlap at this scale. We adopted Figure1 of* [*https://doi.org/10.1371/journal.pntd.0004813*](https://doi.org/10.1371/journal.pntd.0004813) *published under CC BY4.0. [35] as the base layer of this map.*

We went through data sheet and table1 again. An error in table1 also corrected.

……………………………………………………

**Reviewers' comments:**

Reviewer #1: Major comments:
While the study focuses on measuring several indexes of indoor air quality (IAQ), the authors attempt to link IAQ with the risk of respiratory infection. The Introduction and Discussion sections heavily emphasize the association between IAQ and "nosocomial infection" or "COVID-19." While highlighting these links is understandable to some extent, the data and analyses presented in this study do not actually support such claims. For example, in line 276, the authors stress that "this is the first nationwide study that assesses the risk of respiratory infection transmission using CO2 levels…," which seems inappropriate unless the authors also evaluated the presence of viruses or bacteria in the indoor air samples or evaluate the association between IAQ and risk of respiratory disease infection.

There is literature showing that CO2 levels are associated with infection risk estimates despite of some short comings (<https://pubmed(.ncbi.nlm.nih.gov/37566374/> ). A systematic review and meta-analysis (doi: 10.1111/ina.12846 ) illustrated that concentrations of airborne microbes measured as aerobic colony count were correlated with CO2 levels in hospitals [our ref.49]. Low-cost CO2 monitor based indoor infection risk monitoring is accepted by past researchers and implemented in some hospitals & schools etc. ([https://pubmed(.ncbi.nlm.nih.gov/37566374/](https://pubmed.ncbi.nlm.nih.gov/37566374/) , <https://pubmed.ncbi.nlm.nih.gov/34181766/> , <https://pubmed.ncbi.nlm.nih.gov/34224708/> & others). Government agencies of certain countries accept this concept (our line 359-363). In the present study we assume that concept as true (we explain it in introduction). Taking that as true, we measured CO2 levels at OPDs & ETUs as a proxy to get an idea of risk of nosocomial infection (including COVID 19) transmission in those places during peak hours. We are not trying to prove the concept of CO2 levels are associated with infection risk estimates in this study. Hence we believe demonstration of presence of viruses or bacteria in the indoor air samples or evaluation the association between IAQ and risk of respiratory disease infection is not essential here. It is outside scope of our study. Nevertheless, to avoid controversies, we changed the sentence to “To our knowledge, this is the first nationwide study that assesses CO2 levels and other IAQ parameters at all teaching hospital OPDs and ETU/A&Es of any country” (line 318-319).

Table 1 and Figure 3 makes it difficult to understand the overall statistical analysis. It is not clear what was the analysis unit for this study. Are the observations for 15 minutes in a single hospital considered as a single unit, or the real time (seconds or minutes averaged) values for each hospital are used for analysis? How was the data averaged? Were there any missing values? Moreover, it is essential to clarify if the sample size was large enough to conduct t-tests. These issues should be carefully addressed in the statistical analysis section, results, and tables to enhance reader understanding.

Please see the revised Materials and methods section in line 172-238. Former figure 3 is now figure2. We visited each hospital 8.15a.m.-12noon (peak hours) and took each reading continuously for 15 minutes. Complying with the recommendations given in the national IAQ guidelines, we took measurements in three days in each hospital. Total of three measurements per day from OPD, ETU and outdoors of each hospital. There were 63 measurements per one round covering all 21 hospitals and total of 189 measurements for the entire study. Each of those 189 measurement consisted of CO2, air temperature, humidity, PM10, PM 2.5,total particles, VOC and formaldehyde levels. There were no missing data in this data set.

We calculated the averages of CO2, PM10, PM 2.5,total particle, formaldehyde and VOC levels for hospital outdoors, OPDs and ETUs by taking the mean and median of readings of the three data sets (63 data points). Please see lines 240-247.

We did not take a sample to represent all hospitals of the country. We selected all 22 hospitals categorized as teaching hospitals by the Ministry of Health for this study. We managed to get consent of administrators of all to obtain data in their premises. But there is no OPD/ETU in one of them to take data. Therefore we took data from 21. Therefore, sample size is not applicable here. There was no missing data in data set of 21 hospitals (kindly see the supplement file for details).Moreover, to our knowledge there is no minimum sample size required to perform a t-test (<https://www.statology.org/minimum-sample-size-for-t-test/#:~:text=There%20is%20no%20minimum%20sample,the%20results%20could%20be%20unreliable>. ). We verified this by discussing with a statistician of the Ministry of Health as well.

The Figures 1 and 2 is not suitable for publication in scientific journals. The resolution of Figure 3 should be increased, accompanied by a proper explanation. If the authors intend to highlight the difference between outdoor and indoor CO2 levels, Figure 3 should also include outdoor CO2 levels for comparison.

Please note that former figure 3 is now figure2. We are sorry but we sincerely cannot understand why figure 1&2 are unsuitable for publication in scientific journals. If it is due to low resolution in the PDF file sent to reviewers, kindly note that it will be corrected (we have submitted high resolution images). Legend of figure 3 is also revised.
Another possibility is that reviewer#1 may be meaning the places of figures are inappropriate. Hence we removed position of figure1 and made figure 1 as the striking image. Striking image or graphic abstract gained popularity in many journals including those of PloS group during last decade. We give similar figure from the same journal as an example <https://journals.plos.org/globalpublichealth/article?id=10.1371/journal.pgph.0002482> .Yet we think place of figure2 (figure 1 now) is appropriate (we could not find a better place for it). There are such figures in published papers. One example: figure1 of PNAS article <https://www.pnas.org/doi/10.1073/pnas.2208742119> another <https://doi.org/10.1186/s12879-019-3707-y> . Figure 3 (now figure 2) is to show location (distribution) of the teaching hospitals in Sri Lanka. Kindly note that our depicting population density in this map has only a simple reason. It is to show that those teaching hospitals are in highly urban areas. Which explains high outdoor CO2 levels we recorded (line 338-344 ). We did not record good enough data to mark outdoor CO2 distribution in Sri Lanka in a map ( a much larger scale study is necessary for that). So we are unable to show that in figure2. However, we guess that, may be reviewer#1 is asking for indoor and outdoor data in same place for comparison. If so, kindly see the table1 for that purpose.

I hope my comments and suggestions will be helpful in refining this manuscript. Please let me know if any further clarification or information is required.

We hope that these answers would address your concerns. Thank you for the time spent trying to improve our manuscript.

………………………………………………………………

Reviewer #2: Plus Factors- Good study design covering PAN-country; honest mentions of limitations, study is good starting point / a foundation for larger longitudinal / cohort studies.
Lacunas: Had great access to hospital but lack covering patient admission and visit insight data, elaboration on probable pollution sources in premises of identified pollutants.

We admit that information on number of patient visits, identifying probable pollution sources in premises of each hospital would be interesting. However, those were/are beyond the scope of this study. We like other researchers, planned and conducted this study considering human, material, time and financial resources we had and our main interest, our scope did not include those objectives. We had ethical and administrative clearance only to visit 3 days and get only outdoor and indoor air data of OPD & ETU. Yes we are pleased to hear that reviewer#2 identifying this as a good foundation. First author is interested in this topic. He may conduct separate further studies later on those aspects. We added a subtopic of “known sources in OPDs and ETUs that generate air pollutants we studied” to our introduction (line 161).

Methodology reference or standardization to be added, monitoring instrument calibrations frequency and use indoors-outdoors to be elaborated,

Please see the revised Materials and methods section in line 172-236. We visited each hospital 8.15a.m.-12noon (peak hours) and took each reading continuously for 15 minutes. Complying with the recommendations given in the national IAQ guidelines, we took readings for three days in one hospital OPD and ETU and outdoors. Total of three readings per day per each hospital. Total 63 readings per one round covering all 21 hospitals and total of 189 readings. During this study (2022/23) our country was undergoing most severe economic and political crisis that reached its zenith during the time of the study hence importing equipment was almost impossible (There were civil disturbances, banks did not release foreign currency even if you own it, imports were severely restricted). In this background, we managed to obtain two sets of brand-new machines calibrated and quality checked at the factory. We used one set for taking readings. After each round (63 readings) we checked for accuracy/reliability of machines by keeping machines used for data gathering and reference machine set 2-3cm apart and simultaneously measuring air quality parameters in an outdoor open space. The readings were in good agreement within ± 10%.

Line no 150 -bus crash accident- no mention of omission/exclusion in present study,

We introduced a new subtopic. Please see line 232.

Fig-3 hospital legend to mention, also the hospital density and/or ratio wrt population,

Please see modified figure 3 (now figure 2) legend. Our best guess is the reviewer is asking for data on how many hospitals per a population unit. There are 22 Teaching hospitals in Sri Lanka for 22million people (line 99). Approx. 1 teaching hospital per a million population. One hospital does not have OPD/ETU to gather data. So we gathered data from 21. However most of them are concentrated in highly urban areas (figure 2). Kindly note that our depicting population density in this map has only a simple reason. It is to show that those teaching hospitals are in highly urban areas. Which explains high outdoor CO2 levels we recorded (line 334-338 ). We initially tried to put a blue dot plus number at each hospitals location and give names of the hospital in legend. But then at small (i.e: postcard size. The map appears this size when published) the map looks very untidy as several dots and numbers get super imposed, fonts cannot read. So we deleted that. We give hospital list separately (line 268-277)

ventilation coefficient and air exchange rate should be part of the study as they mentioned the importance and consideration in placing monitors, lack in analysis, correlation, and interpretation of parameters other than CO2, or else remove those parameters,

Kindly note that estimations of ventilation coefficient and air exchange were not in our objectives. All OPDs and 10out of 21 ETUs were naturally ventilated. Estimation of those 10 air-conditioned were beyond our objectives/resources. However we added this to the manuscript.”… taking the initiative to install standard air filtration and central ventilation systems at OPDs and ETUs of teaching hospitals at earliest possible time would benefit Sri Lankan public [23,31,34,43,45]. Baseline air changes rate per hour of the ventilation system can be estimated considering the volume of the building, WHO recommended ventilation rate per occupant and average number of occupants [6]. The system can be coupled with a CO2 monitor to do fine tuning of the ventilation depending on CO2 levels to make the process efficient. There are other cheaper bridging solutions to be considered such as the use of portable home air purifiers when PM levels are unusually high coupled with PM level monitoring” [50]..(line 463-471).

Please note that there are recently published studies in good journals (like DOI: 10.1007/s11356-021-13162-8 ) where they studied some of the IAQ parameters we studied, in a few hospitals but to a greater depth. But like us ventilation coefficient and air exchange were not analysed. Hence we think there is no need to remove parameters other than CO2 from the manuscript.

literature review and elaborative discussions in respective sections will have value addition.

We have modified especially the introduction and discussion sections keeping this idea in mind. We hope that the current version of the manuscript would satisfy your requirements.

We hope that these answers would address your concerns. Thank you for the time spent trying to improve our manuscript.

………………………………………………….

Reviewer #3: The article Titled “ Respiratory Infection Transmission Risk and Indoor Air Quality at Outpatient Departments and Emergency Treatment Units of Sri Lankan Teaching Hospitals” is a good attempt to evaluate the air quality in hospitals and medical care facilities. However in its present form it looks a little jumbled up and the authors need to sort out the sectioning to bring out the clarity. Furthermore, the following comments must also be addressed to enhance the quality of the article

The manuscript was extensively revised. Some new sub-headings were added (sectioning was improved) to enhance clarity. We hope that this would address your concerns.

Abstract:
1. Define abbreviations

Implemented. Please see revised abstract.

2. Define all the measured parameters

Implemented. Please see revised abstract. However we did not define μg/m^3^ and mg/m^3^ not to exceed word count ceiling and as such standard measurement values are understood by almost all who read scientific literature. Moreover, the journal website says “Avoid specialist abbreviations”. We think those two are general not specialist abbreviations.

3. Which particular volatile organic compounds were measured?

We measured total volatile organic compounds. Yes there are many of them. But total volatile organic compounds is a standard term used by many similar papers. Here VOC means total volatile organic compounds detected as VOC by the Temtop LKC-1000S+ second generation professional machine. Please see line 130 (The literature that comes with machine and that company website do not give names of individual compounds). We measured formaldehyde levels separately. We think it is more appropriate to give this information outside abstract (word count limit). We give this information in line 127-131 in the introduction.

4. Why COVID-19 is mentioned as a keyword, although its nowhere mentioned in the abstract.

We (like other authors) selected most important messages within word count ceiling for the abstract. But we mention about COVID-19 several times (about 20) in the manuscript (it is also important). Our study would be important and interesting to those who are keen to prevent COVID-19 and similar infections in hospital settings. Considering all we included COVID-19 as a keyword.

Introduction
1. There is no need to define CO2 again

We agree. It was removed.
2. The introduction is long and disjointed. The authors must make it short and relevant to the theme of the article.

We have re-organized the introduction and subheadings of that (added new subheadings).

Our potential audience is diverse but mainly doctors and other healthcare workers. We think keeping details will be useful to the majority. Some parts will be more useful to one segment of the readership while other parts to others. We had to add certain information as suggested by 4 reviewers. Hence we did not shorten the introduction by deleting significantly. We like to explain our point. Doctors in environmental health field and other experts in environmental health field are familiar with PM2.5, PM10, VOC and how they affect human health etc. To our knowledge there are many healthcare workers including very senior doctors in clinical medicine (i.e.: working at OPDs, ETUs) who are not fully familiar with the current knowledge of that aspect. The information given in the introduction will help to understand rest of the article to those who are unfamiliar. There are sub-headings in the introduction. Readers can read sub-headings only, and those who are familiar with a sub-heading can skip that section and save time. We have seen even many senior doctors who are not familiar with current concepts of respiratory infection transmission. For example: In 2020 even then director general of health services of Sri Lanka & most others believed that airborne transmission is negligible (he said so in several televised meetings, did not wear masks in early 2020) for COVID-19. At international level, the director general of the WHO also said so. This situation has changed now. But even now (2023) there are some healthcare workers we know, who are not familiar with current knowledge of respiratory infection transmission. Potential readers include other professionals like building and ventilation engineers, experts in chemistry and architects etc. Our simplified description will help all of them to clearly understand the rest of the article. Those who know this well, can easily skip that sub-heading. All in all we think our modified arrangement would be useful to the majority of our readers.

Material and Methods
1. On which basis the hospital selection was done

We have selected all teaching hospitals (categorized as teaching hospitals by our Ministry of Health) of the country for our study. Because, the largest and most important hospitals of Sri Lanka are among them. During 2019, there were 5.8 million patient visits to OPDs in teaching hospitals of Sri Lanka. Out of 22 teaching hospitals one does not have OPD/ETU. Kindly see lines 99-101, 176-189.

2. Instrumentation is not adequate

We have added more details. Please see lines 214-231.

3. The sample size was not adequate.

We did not took a sample. We selected all 22 hospitals categorized as teaching hospitals by the Ministry of Health for this study. We managed to get consent of administrators of all to obtain data in their premises. But there is no OPD/ETU in one of them to take data. Therefore we took data from 21. Kindly note that we did not select 21 hospitals as a sample of all hospitals of Sri Lanka.

Results and Discussion
1. Some higher order statistics must be implemented to represent the results

Our main objective was to get a basic idea of indoor CO2 levels at OPDs and ETUs of our teaching hospitals. Our secondary objective was to get a basic idea of other IAQ parameters. We prefer to present our findings in a simple and understandable way to be understood by most readers (especially healthcare workers who are not experts in advanced statistics who will form the bulk of our readers). We did analysis and presented our findings keeping those ideas in back of the mind and we wrote discussion based on them. We did analysis thinking of our overall objective. Most past authors of similar papers have studied one or few hospitals in more depth. Our approach is different. We studied all teaching hospitals of the country but in less depth. Some past authors (eg: DOI:10.4103/2230-8229.128778) have estimated correlations between Temp and pollutant levels etc using different statistics. However, their study design, main objective etc are different from ours. Because we present complete measurement set as a supplement file interested readers can extract additional information from that as well.

After thinking we added average PM 2.5 and PM10 levels of ETUs with and without air-conditioning for analysis (line 296-298). We cannot think of another analysis that would improve value of our paper significantly in the aforesaid context. If the reviewer seriously think another calculation is necessary, if you can kindly tell us what specific analysis you need with reason and details we are like to do that to our best ability.

2. Correlate the findings with the previous literature.

There are no past studies of IAQ parameters of hospital OPDs/ETUs of Sri Lanka for comparison. There are past studies from Sri Lanka and other countries on IAQ of homes. But in homes cooking (and sometimes animals) add CO2, CO, particulate matter to air unlike in hospital OPDs/ETUs of Sri Lanka (line 52-54). They are different scenarios that are not comparable well. But we mention results of a VOC study from Sri Lanka. Our study may be the first study from anywhere that study IAQ parameters of a section of all teaching hospitals of the whole country. However we compare our results with results of similar (comparable) past studies from hospitals of India,Lebanon, Bangladesh etc (line 493-523). We have added a new sub-heading (line 493). Also we compare related previous literature in discussion. For example: line 455-458

3. The health risk assessment of these pollutants must be done.

We have added a new subtopic on this matter. Please see lines 133-159.In discussion we propose methods to reduce health risks (line 378-388,400-402, 463-471)

We hope that these answers would address your concerns. Thank you for the time spent trying to improve our manuscript.

………………………………………………..

Reviewer #4: Although, the paper is discussing the indoor CO2 levels which will serve as a proxy for representing the ventilation levels, a simple calculation of Air changes per hour (ACH) in hospitals with manual ventilation would provide a strong foundation for designing efficient ventilation systems.

The term “manual ventilation” is used in health/ medicine to mean giving breathing support or artificial ventilation to a patient manually. We guess that the reviewer#4 here means mechanical ventilation of buildings. We have revised discussion and added the following.

“..taking the initiative to install standard air filtration and central ventilation systems at OPDs and ETUs of teaching hospitals at earliest possible time would benefit Sri Lankan public [23,31,34,43,45]. Baseline air changes rate per hour of the ventilation system can be estimated considering the volume of the building, WHO recommended ventilation rate per occupant and average number of occupants [6]. The system can be coupled with a CO2 monitor to do fine tuning of the ventilation depending on CO2 levels to make the process efficient. There are other cheaper bridging solutions to be considered such as the use of portable home air purifiers when PM levels are unusually high coupled with PM level monitoring” [50]..(line 463-471).

1.	L-193. Use the word “measurements” instead of the word readings and replace the word readings with measurements throughout the manuscript. Implemented.

2.	L 256-257, Provide the reason for high levels of pollutants since it is mentioned only as unusually high. Give the reason for these unusually high levels of pollutants.

We added following to the discussion (line 314-317). On December 12,2022 PM 2.5 and PM10 levels of both OPD and ETU of Chest hospital- Welisara were very high. Corresponding outdoor levels were also high but the gap between indoor and outdoor levels was large. Therefore we think high outdoor levels plus additional reasons not noticed by us contributed to this very high indoor particulate matter levels.

3.	L 333 – 335, check the grammar and rewrite the sentence providing the same meaning. Changed to: Outdoor PM 2.5 levels in Sri Lanka and in neighboring South Asian countries are higher than WHO ceilings. The situation in Sri Lanka is better than her neighbors [20,39,40].

4.	L 386-390, Provide the reason for high CO2 Levels.

We believe that was a result of inadequate ventilation in relation to the population inside.(kindly see line 422-423)

5.	L 394 – Check grammar and rewrite the sentence.

It was changed to: Average PM 2.5 and PM10 of both outdoor and indoor air were higher than the current WHO recommended levels. However, they were lower than national ceilings.(please see line 431-432)

We hope that this would address your concerns. Thank you for the time spent trying to improve our manuscript.
