# Supplementary material for: Respiratory infection transmission risk and indoor air quality at outpatient departments and emergency treatment units of Sri Lankan teaching hospitals
Source: PLOS Glob Public Health. 2024 Feb 26;4(2):e0002862. doi: 10.1371/journal.pgph.0002862 (PMC10896534; doi:10.1371/journal.pgph.0002862)
Supplement: Attachment — Submitted filename: Response to Reviewers-R2-December 2023.docx [file pgph.0002862.s005.docx]

**PGPH-D-23-01122
Respiratory Infection Transmission Risk and Indoor Air Quality at Outpatient Departments and Emergency Treatment Units of Sri Lankan Teaching Hospitals.
PLOS Global Public Health**

**The rebuttal letter that responds to each point raised by the editor and four reviewer#1- R2.**

Dear Dr. Naveen Puttaswamy, Ph.D,

Thank you for the email dated December 22, 2023.

The revised clean file version of the manuscript and the marked-up version with alterations highlighted are also submitted with this.

To enhance clarity, our responses are in green color fonts.

We hope we have satisfactorily addressed all the comments/concerns reasonably and hope that our paper may now be suitable for publication in your journal.

Dr.NDB Ehelepola (Corresponding author)

……………………………………………………

**Reviewers' comments:**

Reviewer #1: The authors stated, "not trying to prove the concept of CO2 levels are associated with infection risk estimates in this study," but the title and introduction of the paper still present the possibility of misinterpretation by readers. Therefore, I would like to reiterate the opinion that an overall modification of the term “proxy” and use of “respiratory infection transmission risk” in this study. Additionally, please re-examine the prerequisites for t-test analysis and present the results together.

In the authors' cited review paper 49, there was a significant association between CO2 and airborne microbial concentration. However, by considering the limited number of relevant papers (n=3) for the meta-analysis and the research design and small sample size of individual studies, the evidence supporting the authors' claim that CO2 levels can be used as a proxy for infection appears insufficient. It is recommended to describe this paper as a descriptive study on indoor air pollution levels in hospitals and just briefly indicate the potential associations with various infections and indoor air quality.

Please review the figures once again and ensure that each is self-explanatory.

We like to explain our response to reviewer#1 in R1 again. In our response, we gave examples of literature showing that CO2 levels are associated with infection risk estimates despite of some short comings and how government agencies of certain countries accept this concept and how some hospitals etc. of certain countries already use CO2 levels as a proxy of respiratory infection risk. Then we wrote “In the present study we assume that concept as true (we explain it in introduction). Taking that as true, we measured CO2 levels at OPDs & ETUs as a proxy to get an idea of risk of nosocomial infection (including COVID 19) transmission in those places during peak hours”. Actually this has been the core theme of our study from the inception. After clearly telling that we assume aforesaid concept as true, responding to the question, we said “We are not trying to prove the concept of CO2 levels are associated with infection risk estimates in this study. Hence we believe demonstration of presence of viruses or bacteria in the indoor air samples or evaluation the association between IAQ and risk of respiratory disease infection is not essential here”. In other words we are telling that we are not trying to prove the concept of CO2 levels are associated with infection risk estimates in this study because we accept that concept based on existing evidence/information and here we use CO2 levels as a proxy to get an idea of risk of nosocomial infection (including COVID 19) transmission in our hospitals.

The reviewer citing our reference 49 says that the evidence supporting the authors' claim that CO2 levels can be used as a proxy for infection appears insufficient. That implies that the reviewer1 is reluctant to believe that indoor CO2 concentration can be used as a proxy of the degree of ventilation and, by extension, as an indicator of the risk of contracting respiratory infections. We like to kindly remind that CO2 levels indicate crowdedness as well. Not only the concentration of pathogens in air but crowdedness (occupancy) also influence respiratory infection transmission [ref8 & others ].

Kindly note that the reference 49 is only one evidence. There are other related references as well [5,8,9,10,11,38,41]. Journal article writers usually give a few references to support one point. We have given 7. Moreover, in the introduction we have 2 subtopics that elaborate our point namely “Current knowledge on how respiratory infections get transmitted and the basis for our use of indoor CO2 levels as a proxy of the risk of contracting respiratory infections” and “Reasons for our study using CO2 levels as a proxy to assess the risk of contracting respiratory infections at Sri Lankan hospitals”. We think that those are good enough to support our point. As the reviewer did not give any reference/s that show indoor CO2 concentration cannot be used as a proxy of the risk of contracting respiratory infections, we searched in the pubmed and google scholar for “indoor CO2 concentration not correlated with respiratory infection risk” on December 23, 2023. There were several more publications to show that indoor CO2 concentration is correlated with respiratory infection risk but not a single paper demonstrating the opposite.

Nevertheless, we understand that this concept is not universally accepted like many other widely practically used concepts in medicine/ public health. To accommodate alternative ideas we added the following paragraph to limitations sub-topic.

Indoor CO2 concentration has been used as a proxy of the degree of ventilation and, by extension, as an indicator of the risk of contracting respiratory infections in that space [5,8,9,10,11,38]. Based on available literature that we mentioned in introduction and in discussion we are convinced that concept is valid [5,8,9,10,11,38,41,49]. Thus we did not attempt to prove that concept again in this study.

We assumed that concept as true and conducted the study although the concept is not universally accepted (line 543-546). Considering all we think we need not to alter the title or the introduction (those say what we mean).

We did not add more to the manuscript to support our idea of “indoor CO2 concentration is correlated with respiratory infection risk” because we already have given a substantial attention to it. We need to have a balance in emphasis given to each of several component of our paper. Giving undue emphasis to this component is not good. However to further support/strengthen our answer we like to mention the following published articles. <https://journals.sagepub.com/doi/full/10.1177/1420326X211043564> ,

<https://pubs.acs.org/doi/abs/10.1021/acs.estlett.1c00183> ,

<https://www.cambridge.org/core/journals/flow/article/monitoring-carbon-dioxide-to-quantify-the-risk-of-indoor-airborne-transmission-of-covid19/245A8FE68DD9C07655B9F25BECE967D2> ,

<https://journals.sagepub.com/doi/full/10.1177/0022034520940288> ,

<https://www.mdpi.com/2071-1050/13/21/12203> ,

<https://www.sciencedirect.com/science/article/pii/S187603412300165X>

For review1 (R1) we responded “to our knowledge there is no minimum sample size required to perform a t-test (<https://www.statology.org/minimum-sample-size-for-t-test/#:~:text=There%20is%20no%20minimum%20sample,the%20results%20could%20be%20unreliable> ). We verified this by discussing with a statistician of the Ministry of Health as well “.Considering reviewers concerns we re-checked literature on this issue. We found some academic articles supporting our view such as <https://doi.org/10.7275/e4r6-dj05> . Nevertheless there are articles recommending higher sample size/lager number like 40 for more reliable results like the paper <https://www.ncbi.nlm.nih.gov/pmc/articles/PMC4744321/> . Only 21 teaching hospitals with OPDs and ETUs are in this country and we have finished data collection long ago. So there is no way of changing number 21. Another test to substitute t test is Wilcoxon signed-ranks test <https://datatab.net/tutorial/wilcoxon-test> . Minimum number (sample) for that test is said to be 16, DOI: 10.1002/sim.7263. We performed Wilcoxon signed-ranks test (using SPSS 23.0) and obtained the same results. That improves trustworthiness of that results. Please see line 286-292 as well.

We have reviewed the figures once again considering your suggestion. We have modified the striking image reducing the count of words in it. We have added details to the legend of figure 1. We think figures 2 and 3 and their legends do not need modifications.

Please see the table1 to see the results summary together (in one place). Results of further analysis can be seen in lines 286-319.

We hope that this would address your concerns. Thank you for the time spent trying to improve our manuscript.
